# The Biological Basis for Enhanced Effects of Proton Radiation Therapy Relative to Photon Radiation Therapy for Head and Neck Squamous Cell Carcinoma

**DOI:** 10.14338/IJPT-20-00070.1

**Published:** 2021-06-25

**Authors:** Li Wang, Piero Fossati, Harald Paganetti, Li Ma, Maura Gillison, Jeffrey N. Myers, Eugen Hug, Steven J. Frank

**Affiliations:** 1 Department of Experimental Radiation Oncology, The University of Texas MD Anderson Cancer Center, Houston, TX, USA; 2 Department of Radiation Oncology, MedAustron Ion Therapy Center, Wiener Neustadt, Austria; 3 Department of Radiation Oncology, Massachusetts General Hospital and Harvard Medical School, Boston, MA, USA; 4 Department of Thoracic-Head & Neck Med Oncology, The University of Texas MD Anderson Cancer Center, Houston, TX, USA; 5 Department of Head & Neck Surgery, The University of Texas MD Anderson Cancer Center, Houston, TX, USA; 6 Department of Radiation Oncology, The University of Texas MD Anderson Cancer Center, Houston, TX, USA

**Keywords:** proton radiation therapy, x-ray radiation therapy, head and neck cancer, biological effect, radiation sensitization

## Abstract

Head and neck squamous cell carcinomas (HNSCCs) often present as local-regionally advanced disease at diagnosis, for which a current standard of care is x-ray–based radiation therapy, with or without chemotherapy. This approach provides effective local regional tumor control, but at the cost of acute and late toxicity that can worsen quality of life and contribute to mortality. For patients with human papillomavirus (HPV)–associated oropharyngeal squamous cell carcinoma (SCC) in particular, for whom the prognosis is generally favorable, de-escalation of the radiation dose to surrounding normal tissues without diminishing the radiation dose to tumors is desired to mitigate radiation-related toxic effects. Proton radiation therapy (PRT) may be an excellent de-escalation strategy because of its physical properties (that eliminate unnecessary radiation to surrounding tissues) and because of its biological properties (including tumor-specific variations in relative biological effectiveness [RBE] and linear energy transfer [LET]), in combination with concurrent systemic therapy. Early clinical evidence has shown that compared with x-ray–based radiation therapy, PRT offers comparable disease control with fewer and less severe treatment-related toxicities that can worsen the quality of life for patients with HNSCC. Herein, we review aspects of the biological basis of enhanced HNSCC cell response to proton versus x-ray irradiation in terms of radiation-induced gene and protein expression, DNA damage and repair, cell death, tumor immune responses, and radiosensitization of tumors.

## Introduction

Squamous cell carcinoma of the head and neck (HNSCC) is the sixth most common cancer worldwide, with 650 000 new cases diagnosed and 330 000 deaths each year [1]. Because HNSCC often presents as local-regionally advanced disease at diagnosis, photon (x-ray)–based intensity-modulated radiation therapy (IMRT), with or without chemotherapy, is a current standard of care [2]. Unfortunately, IMRT often has severe short- and long-term treatment-related toxic effects (eg, dysgeusia, dysphagia, aspiration, mucositis, soft tissue necrosis, osteoradionecrosis, and cranial neuropathy) [3, 4], especially when used for oropharyngeal cancers. Two biologically and clinically distinct types of oropharyngeal squamous cell carcinoma (SCC) have been identified, one related to tobacco and alcohol consumption and the other associated with human papillomavirus (HPV) infection. HPV-positive oropharyngeal SCC is more common among individuals younger than 50 years, who now constitute more than 70% of new oropharyngeal SCC cases [2, 5]. Notably, the 5-year overall survival rate after IMRT is greater than 80% for patients with HPV-positive oropharyngeal SCC but is only about 40% for patients with HPV-negative disease [6]. Thus, new strategies are urgently needed to intensify treatment approaches to improve outcomes for patients with poor-prognosis HPV-negative oropharyngeal SCC, as well as de-intensified treatment approaches to minimize or eliminate radiation-related toxicity in patients with HPV-positive disease, to improve tumor control and quality of life for all patients with oropharyngeal SCC [7].

Although the proximity of numerous critical organs and structures in the head and neck region makes IMRT challenging for treating HNSCC, the more precise technique of intensity-modulated proton therapy (IMPT) is a promising alternative because, relative to photons, protons allow highly conformal dose distributions to tumors with a considerable reduction of the integral dose to normal tissue [8], fewer treatment-related side effects [9–11], and presumably comparable disease control. This capability for highly conformal doses allows escalation of doses to tumors with simultaneous de-escalation of the dose to normal tissues. Moreover, x-ray beams have low linear energy transfer (LET), whereas proton beams, especially at the distal edge of the spread-out Bragg peak (SOBP), have higher LET. High-LET proton beams are known to have higher relative biological effectiveness (RBE) than low-LET x-ray beams [12, 13] through several mechanisms, including differences in their effects on DNA damage and repair, among others [14–16]. Thus, in addition to the physical advantage of dose deposition patterns, IMPT may also have distinct biological advantages over IMRT for HNSCC. However, relatively little information has been published on these potential biological advantages.

To fill this gap in information, we review and highlight current knowledge of the presumed enhanced biological effectiveness of proton versus x-ray radiation in HNSCC and its possible mechanisms, which include differences in the molecular-level effects of protons versus x-rays on gene and protein expression, DNA damage and repair, cell death mechanisms, tumor immune responses, and sensitization of HNSCC to radiation. Our hope is that this overview of the biological advantages of IMPT versus IMRT will ultimately be beneficial in terms of facilitating treatment intensification for HPV-negative HNSCC and treatment de-intensification for HPV-positive HNSCC.

## Contributors to the Enhanced Biological Effects of Proton versus Photon Radiation in Head and Neck Cancer

### Relative Biological Effectiveness of Protons versus Photons in HNSCC

The biological effects of the various types of radiation are determined by the energy deposition pattern of the specific beams. Thus, low-LET photon beams and high-LET proton beams do not produce equal biological effects at the same dose (reviewed in Wang and Frank [16]). The RBE of proton radiation is defined as the ratio of the photon dose to the proton dose required to produce the same level of biological effect, such as cell killing or DNA damage [17]. In current clinical practice, the RBE value of proton versus photon radiation has been assumed to be 1.1 [18, 19]. This RBE value, which is greater than 1.0, indicates that protons have enhanced biological effects, compared with photon radiation. However, RBE values also depend on physical aspects of the cells or tissues that the beams traverse [19]. Current evidence (Table), although limited [20, 21], supports our contention that protons have enhanced biological effects relative to photons in HNSCC.

**Table.** Summary of proton versus photon radiation RBE experiments in head and neck squamous cell carcinoma cell lines.

**Cell line**	**Radiation type and dose**	**Biological endpoint**	**RBE**	**Reference**
SqCC/Y1	6-MV x-ray (ref)			
	200-MeV proton			
	2 Gy	Clonogenic survival	1.18	20
	6 Gy	Clonogenic survival	1.08	
HN5	6-MV x-ray (ref)			
	200-MeV proton			
	2 Gy	Clonogenic survival	1.19	20
	6 Gy	Clonogenic survival	1.06	
MDA686Tu	6-MV x-ray (ref)			
	200-MeV proton			
	2 Gy	Clonogenic survival	1.32	20
	6 Gy	Clonogenic survival	1.22	
UMSCC-47	6-MV x-ray (ref)			
	200-MeV proton			
	2 Gy	Clonogenic survival	1.15	20
	5 Gy	Clonogenic survival	1.09	
UCPI-SCC-154	6-MV x-ray (ref)			
	200-MeV proton			
	2 Gy	Clonogenic survival	1.17	20
	6 Gy	Clonogenic survival	1.12	
UCPI-SCC-152	6-MV x-ray (ref)			
	200-MeV proton			
	2 Gy	Clonogenic survival	1.19	20
	6 Gy	Clonogenic survival	1.11	
HN5	6-MV x-ray (ref)			
	200-MeV proton			
	4 Gy	Persistence of DSBs (53BP1 foci)	1.54	20
	Persistence of DSBs (NCA)	1.46		
UMSCC-47	6-MV x-ray (ref)			
	200-MeV proton			
4 Gy		Persistence of DSBs (53BP1 foci)	1.42	
	Persistence of DSBs (NCA)	1.28		
CAL33	6-MeV x-ray (ref)			
	63-MeV proton			
	1, 2, 4, or 6 Gy	Clonogenic survival	1.10	21

**Abbreviations:** RBE, relative biological effectiveness; DSBs, DNA double-strand breaks; 53BP1, p53-binding protein 1; NCA, neutral comet assay.

Note: In the study of Wang et al [20], 200-MeV clinical proton beams were used to deliver radiation to an 18×18-cm field, with cells positioned at the center of the field. Cells were irradiated and doses measured in the middle of the spread-out Bragg peak (see Figure 1). In the study of Lupu-Plesu et al [21], proton irradiations were carried out with 63-MeV beams.

**Figure 1.**
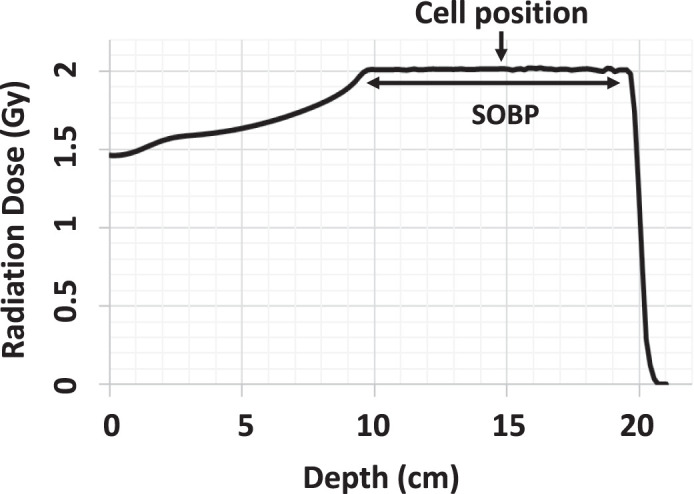
Positioning of cell samples at the middle of the SOBP of proton irradiation [20]. The depth was generated by using water-equivalent material. Abbreviation: SOBP, spread-out Bragg peak.

In one study that used clonogenic survival as an endpoint [20], the RBE values for protons versus photons were found to vary in 3 HPV-positive and 3 HPV-negative HNSCC cell lines, and the variation depended on both cell line and radiation fraction size. When the cell samples were positioned at the middle of the SOBP (Figure 1), the RBE values were found to be higher for protons than for photons (ie, the biological effect was enhanced) in all 6 HNSCC cell lines tested (RBEs all >1.06), and single fractions of 2 Gy each (as used in clinical situations) had greater RBE values (range, 1.15-1.33) than did single 4-Gy or 6-Gy fractions. Similarly, when the chosen endpoint was persistence of DNA double-strand breaks (DSBs) (representing unrepaired DNA damage), at 24 hours after a single 4-Gy dose of radiation, protons were found to have induced greater effects than x-rays, with neutral comet assays showing a 1.42-fold increase in persistent 53BP1 (p53-binding protein 1) foci and a 1.28-fold increase in unrepaired DSBs in all cell lines tested [20]. Similarly, in another study that used clonogenic survival as an endpoint, protons were found to kill HNSCC cells more efficiently than x-ray radiation, with an RBE value of 1.1 [21]. However, no enhanced biological effect was observed for protons versus photons when the HNSCC cells were exposed to multiple fractions totaling 8 Gy, when the endpoint was number of viable cells at 96 hours after irradiation [21].

These findings indicate that protons have enhanced biological effects relative to photons in HNSCC. Further studies are needed to clarify the nature of the biological advantage of using proton versus photon radiation in terms of treatment gains.

### LET-Based Optimization of Proton Treatment Plans for HNSCC

Emerging evidence has revealed another notable feature of RBE for protons versus photons that could be exploited in radiation treatment plans: a significant variation in LET values across the irradiated volume. As the RBE increases with increasing LET, the RBE value tends to increase along the beam path to the end of the SOBP in a single-field uniform dose. Plans for proton treatments could have an additional advantage over plans for photon therapy in HNSCC, in that optimizing LET with a multifield uniform dose in IMPT might enhance the biologically effective dose to the tumor and reduce that to the organs at risk [12, 13,18], presumably enhancing the tumor response while reducing normal-tissue damage [22]. This concept is being exploited for planning purposes by using analytical methods or Monte Carlo simulations to precisely predict LET along the radiation beam in patients with HNSCC [23]. These predictions have been incorporated into an optimized method to avoid accidentally placing critical normal structures in high-LET regions, instead ensuring that the tumors are there to avoid compromising target coverage. This distal-edge avoidance-guided optimization method (DEAOpt) was used to create proton treatment plans for 2 patients with HNSCC, which were then compared with treatment plans based on conventional dose-based optimization. As expected, the DEAOpt plans led to reduced RBE “hot spots” in critical structures and increased the RBE in the tumors.

**Figure 2.**
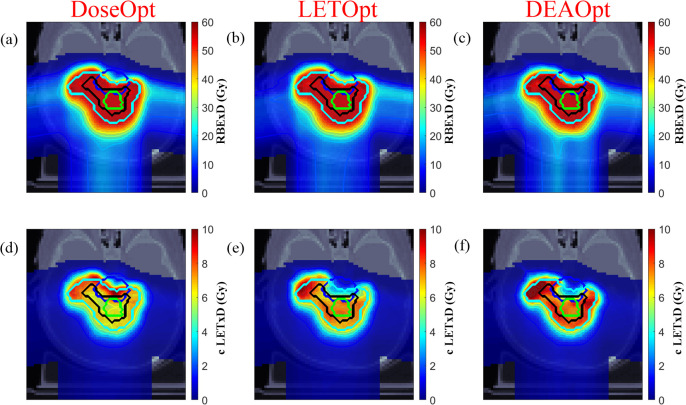
Proton treatment plans for a patient with a brain tumor derived from conventional DoseOpt (left), LETOpt (middle), and DEAOpt (right) method were compared. The top row (a, b, and c) shows the dose distributions (based on a constant RBE of 1.1). The bottom row (d, e, and f) shows the distributions of cLETxD, that is, the LET-weighted dose scaled by the factor c (the value of c = 0.04 μm/keV). Contours for the gross target volume are shown in green; the clinical target volume, in black; the planning target volume, in cyan; and the brainstem, in blue. Dose distributions were similar for the LETOpt and DEAOpt plans, and brainstem sparing was better in the LETOpt plan than in the DoseOpt plan (figure reproduced with permission from Bai X, Lim G, Grosshans D, Mohan R, Cao W. A biological effect-guided optimization approach using beam distal-edge avoidance for intensity-modulated proton therapy. Med Phys. 2020;47:3816–25). Abbreviations: DEAOpt, distal-edge avoidance-guided optimization; DoseOpt, dose-based optimization; LETOpt, LET-incorporating optimization; RBE, relative biological effectiveness.

Although considerably more research is needed, this preliminary evidence suggests that protons can have greater biological effects than photons in HNSCC and that treatment plans can be optimized to match regions of high LET with tumor targets and away from normal tissues (Figure 2). In other words, proton radiation may have the potential for intensified doses to tumors with de-intensified doses to normal tissues, both of which would have effects favorable to those that are possible with photon radiation. Moreover, the relationship between RBE, LET, and radiation fraction size suggests that altered fractionation schedules (eg, hyperfractionation) may well lead to further treatment benefits. Further research in this area is warranted.

## Mechanisms Underlying the Enhanced Biological Effects of Proton versus Photon Radiation in Head and Neck Cancer

### Proton- versus Photon-Induced Changes in Gene and Protein Expression in HNSCC

To date, little is known of whether protons elicit patterns of protein and gene expression in HNSCC cells that are different than those elicited by photons [21, 24], although differences in these patterns may offer valuable clues to understanding the mechanisms underlying the enhanced biological effects of protons over photons in HNSCCs.

One attempt to address this question involved exposing 2 HPV-negative and 2 HPV-positive HNSCC cell lines to a single 4-Gy dose of protons or x-rays and using reverse-phase protein array analysis to simultaneously evaluate the expression of 175 proteins involved in a variety of signaling pathways, including DNA damage response (DDR), cell cycle regulation, apoptosis, senescence, cell proliferation, immune response, and metabolism [24]. At 1 hour, 4 hours, or 24 hours after irradiation, protons prompted the expression of more and higher levels of proteins related to DDR (eg, catalytic subunit of DNA-dependent protein kinase [DNA-PKcs] and H2AX [pS139]), cell cycle arrest (eg, CHK1.pS345), anti–cell growth, and anti–cell proliferation than did exposure to x-rays; on the other hand, x-rays led to expression of more and higher levels of proteins associated with cell cycle progression (eg, cyclins B1 and E1), cell proliferation (eg, epidermal growth factor receptor, activated form of PI3K), and cell growth than did protons. Moreover, protons and photons elicited different expression patterns of several immune response–related proteins. Collectively, these findings suggest that protons tend to induce a pattern of pro–cell-killing protein expression in HNSCC cells relative to x-rays, which may explain part of the observed enhancement of HNSCC cell death by protons over x-rays [20].

Another group in France reported that exposing HNSCC cells to proton or x-ray irradiation led to different expression profiles of genes involved in angiogenesis, cell proliferation, and metastasis [21]. Specifically, exposure to protons led to reduced expression of genes related to proliferation, angiogenesis, and lymphangiogenesis. Another group in Japan found that expression of the vascular endothelial growth factor C (VEGF-C) was linked to unfavorable prognosis in patients with HNSCC [25]. The French group confirmed that although both proton and x-ray radiation activated the *VEGFC* promoter in HNSCC cells, cells exposed to protons showed lower levels of gene and protein expression of VEGF-C relative to x-rays, and that irradiation with x-rays prompted a more aggressive tumor phenotype in vivo, with increased angiogenesis as well as overexpression of PLK1 (polo-like kinase 1, an inhibitor of apoptosis) or TRF2 (telomeric repeat binding factor 2), which has been linked with poor prognosis in patients with HNSCC [26, 27]. Other genes found to be expressed at higher levels after x-ray irradiation include those involved in metastasis, angiogenesis, and epithelial-mesenchymal transition (eg, *MMP2*, *MMP9*) [28]. These observations suggest that protons are less likely to result in angiogenesis, lymphangiogenesis, or metastasis than are x-rays in HNSCC [21].

Collectively, these findings indicate that proton and photon radiation cause different patterns of change in HNSCC cell lines in several signaling pathways (eg, cell proliferation, cell cycle regulation, DNA damage repair, angiogenesis, lymphangiogenesis, or metastasis). These differences further suggest that protons may have enhanced biological effects relative to photons, particularly for favorable-prognosis (ie, HPV-positive) HNSCC. Further studies are needed to clarify and extend these findings.

### Proton- versus Photon-Induced DNA Damage and Repair in HNSCC

Radiation-induced DNA DSBs and DDR are crucial determinants of the fate of irradiated cells. Failure to repair DNA DSBs can lead to gene mutations, chromosomal aberrations, cell death, or malignant cell transformation [14, 15]. The mechanisms underlying x-ray–induced DDR have been studied extensively. However, much less is known of how protons affect DNA DSBs, and the mechanisms underlying DDR also remain to be elucidated (summarized in Wang and Frank [16]).

Because higher-level (ie, clustered) DNA damage is unlikely to be repaired successfully compared with simpler single-strand or DSBs, an irradiated cell's fate is also determined by the complexity of the damage to the DNA. Mathematical modeling has shown that the complexity of DNA damage increases with increases in LET; hence at or around the Bragg peak, protons cause more complex DNA damage than x-rays [14, 29]. This phenomenon has been verified by the persistence of DNA DSBs after proton versus photon irradiation in several cancer cell types [16], including HNSCC [20, 30]. As described above in the section on RBE, protons caused more unrepaired DNA DSBs than did x-rays in HPV-positive and HPV-negative HNSCC cell lines [20], as indicated by numbers of 53BP1 foci at 24 hours and by neutral comet assay tail moment. Another group found similar results for 2 HPV-negative HNSCC cell lines [30].

As for DDR, the 2 major types of damage repair are homologous recombination (HR) and nonhomologous end-joining (NHEJ). Although considerable evidence exists to indicate that photon-induced DNA DSBs are repaired mostly via NHEJ, how proton-induced DNA DSBs are repaired remains unclear [16, 31]. The sole report to date comparing the mechanisms of proton- versus photon-induced DDR in 2 HPV-positive and 2 HPV-negative HNSCC cell lines indicated that both HR and NHEJ pathways were activated after proton irradiation [24].

In summary, proton radiation seems to cause more persistent DNA DSBs in HNSCC cells than photon radiation, which in turn suggests that protons have enhanced biological effectiveness relative to photons in HNSCCs. Which DSB repair mechanisms are used after proton versus photon radiation remains unclear. Future studies on these topics may serve as the basis for selecting molecular targets to improve the response of HNSCC to proton or photon radiation.

### Mechanisms Underlying Proton- versus Photon-Induced HNSCC Cell Death

The failure to successfully repair photon-induced DNA damage eventually leads to cell death through pathways including apoptosis, necrosis, autophagy, mitotic catastrophe, or senescence (summarized in Wang and Frank [16]). The mechanisms by which protons induce cell death, especially in HNSCC cells, remain to be established.

Our own investigation of cell death mechanisms after a 4-Gy dose of protons versus photons in 2 HPV-positive and 2 HPV-negative HNSCC cell lines [32] revealed mitotic catastrophe to be the predominant mechanism of cell death at 4, 24, 48, or 72 hours after either type of radiation. Notably, protons led to greater amounts of mitotic catastrophe than x-rays. Another important x-ray–induced type of cell death, cellular senescence [16], was also found after proton or x-ray irradiation, again with protons producing larger percentages of senescent HNSCC cells at 4 days or 6 days after exposure [32]. Protons have also been shown to kill cells by apoptosis, with some studies suggesting that protons induce more apoptotic cells than photons [16]. Our own findings indicate that x-rays and protons both led to only limited apoptosis in HNSCC cells, and that the proportions of apoptotic cells were similar after either type of radiation [32]. We further found that protons and x-rays induced similar levels of HNSCC cell necrosis [32], which usually occurs after large doses of photon radiation [16].

To summarize, although few studies have been done, protons seem to produce higher proportions of HNSCC cells undergoing mitotic catastrophe and senescence than do x-rays, implying again that protons may have greater biological effects than photons in HNSCC. Further studies in this area are needed to establish a rationale for combination treatments that target cell-death pathways to enhance the effectiveness of proton or photon therapy for HNSCC.

### Effects of Protons versus Photons on Immune-Related Responses in HNSCC

Radiation is known to have both immunosuppressing and immunoactivating effects [16]. Indeed, the immunoactivating effects of photon radiation in combination with immunotherapy have made this an increasingly promising approach for improving cancer treatment outcomes [33] and is under extensive study [16]. Given the differences in physical and biological features of protons and photons, another major question to be answered is whether protons are superior to photons in combinations with immunotherapy.

Photon radiation is known to induce an “in situ autovaccination” effect, a means of immunoactivation in which various small molecules are expressed on tumor cells after radiation-induced cell death; these molecules include DAMPs (damage-associated molecular patterns), calreticulin, HMGB1 (high mobility group box-1), and ATP (reviewed in Wang and Frank [16]). Although very few such studies have been done on proton radiation to date [34, 35], similar phenomena have been reported for tumor cells exposed to protons. Photon radiation is also known to have nontargeted immunoactivation effects, including the bystander and abscopal effects. However, whether protons elicit these effects is largely unknown [16], and the mechanisms by which photons or protons lead to immunoactivation in HNSCCs are unknown.

With regard to immunosuppressive effects, photon radiation can directly kill immune cells or it can indirectly suppress their function (reviewed in Wang and Frank [16]). Because the extent of radiation-induced immunosuppression depends on the radiation field size and whether lymph nodes are included in those fields [36], protons are likely to have less of an immunosuppressive effect than photons because smaller amounts of normal tissues, including immune cells (eg, T lymphocytes), are exposed to radiation. Another mechanism by which photons induce immunosuppression is the induced secretion of cytokines (eg, interleukin [IL] 10, IL-6, IL-8, transforming growth factor β, prostaglandin E2) by the radiation-damaged cells that suppress immune cell functions (reviewed in Wang and Frank [16]). Although little work has been done on protons in this regard, proton radiation has been reported to reduce IL-6 and IL-8 levels in both in vitro and in vivo studies [37, 38]. IL-6 expression in both tumors and normal tissues has been linked with unfavorable treatment outcomes in patients with HNSCC [39]. Another study of 2 HNSCC cell lines revealed that protons led to higher *IL6* gene expression but lower *IL8* gene expression relative to photon radiation [21]. Another group showed that protons downregulated the expression of CCL2 (C-C motif chemokine ligand 2), a proinflammatory cytokine linked with HNSCC progression [40], relative to photons, in HNSCC cells. Moreover, it has been suggested that proton radiation differs from photon radiation in the reprogramming of M1/M2 macrophages so as to enhance proinflammatory and antitumoral functions [41].

Inhibitors targeting the T-cell function inhibitors PDL1 (programmed death-ligand 1) and CTLA4 (cytotoxic T-lymphocyte–associated protein 4) have recently shown some success in clinical applications [42, 43]. One rationale for combining immune checkpoint inhibitors with photon radiation is that photons upregulate PDL1 expression (reviewed in Wang and Frank [16]). Whether protons also affect PDL1 expression is yet to be determined. High expression of PDL1 in primary tumors has been linked with unfavorable prognosis after photon radiation treatment in patients with HNSCC [44, 45]. Encouragingly, several HNSCC cell lines showed lower expression of both the *PDL1* gene [21] and the PDL1 protein [24] after exposure to protons versus x-rays. Conversely, CTLA4 protein expression was higher in HNSCC cell lines after exposure to protons versus x-rays [24].

Collectively, the very few studies done to date suggest that proton and photon radiation may have similar immunoactivation effects, but protons may have less of an immunosuppression effect. Because protons and photons seem to evoke the expression of different patterns of inflammatory cytokines and T-cell function inhibitors in HNSCC cells, the potential benefits of combining different immune checkpoint inhibitors with proton or photon radiation need further study.

### Enhancement of Head and Neck Cancer Response to Proton versus Photon Radiation

To improve treatment gain in HNSCCs, the most important strategy is to identify effective treatment approaches that can improve tumor response while minimizing treatment-related toxicity. One such approach, targeting DDR signaling pathways in combination with photon radiation, has been studied extensively [46, 47], but studies of combinations of DDR inhibitors and proton radiation for HNSCC have been rare, and whether protons enhance the effects of such inhibitors remains to be seen.

Inhibitors of poly (ADP-ribose) polymerase [PARP] are well known to sensitize HNSCC to photon radiation [46, 48]. However, only 1 study thus far has shown a radiation enhancement effect for a PARP1/2 inhibitor, niraparib, on the response of HNSCC cells to both proton and photon radiation [48]. In that study, the potential sensitization effect of using niraparib to block DDR was investigated in 3 HPV-positive and 2 HPV-negative HNSCC cell lines exposed to proton or photon radiation [48]. When clonogenic survival and unrepaired DNA DSBs were used as endpoints, niraparib was found to sensitize all of these HNSCC cell lines to both forms of radiation, but to slightly different extents: niraparib improved the proton versus x-ray RBE values by about 10% in the HPV-positive cell lines and by about 3% in the HPV-negative cell lines, which were tested at a surviving-cell fraction of 0.1. Also, giving niraparib concurrently with protons caused more persistent DNA DSBs than giving niraparib concurrently with photons. Another PARP inhibitor, olaparib, was also found to enhance the response of 1 HPV-negative HNSCC cell line to high-LET protons [49]. Additional studies are needed to establish the value of PARP inhibitors in improving the response of HNSCC to protons versus photons in both in vivo and clinical settings.

Targeting key DDR protein kinases such as ATR (which targets HR), ATM, and DNA-PKcs (which target NHEJ) has also been effective in enhancing the response of HNSCC cells to photon radiation [46], and some of these agents are currently in clinical trials. However, only 1 study to date has reported on whether these inhibitors sensitize HNSCC to protons as opposed to photons [50]. In that study, ATR, ATM, and DNA-PKcs inhibitors were tested for potential enhancement effects, with the endpoints being clonogenic survival and 3D spheroid growth rate, in HPV-positive and HPV-negative HNSCC cells exposed to photon or proton radiation. Significant enhancement effects for photon radiation were noted, especially use of a DNA-PKcs inhibitor with HPV-negative HNSCC cells; enhancement effects were similar for DNA-PKcs and, to a lesser extent, for an ATM inhibitor, for proton radiation. These findings suggest that these inhibitors could be used in combination with proton or photon radiation to enhance treatment efficacy in HNSCC, especially the relatively radioresistant HPV-negative tumors.

In sum, much additional work is needed on the potential use of DDR inhibitors to enhance the responsiveness of HNSCC to proton radiation. The information gathered to date on the biological effects of protons versus photons in HNSCC points to the need for additional investigations combining proton radiation with molecular or immune targeted therapy (eg, antiangiogenic VEGF-C, depletion of USP6 [ubiquitin specific peptidase 6], anti-immune checkpoints) to enhance the antitumor effects of proton radiation, with the ultimate goal of improving tumor control while minimizing treatment-related toxicity for patients with HNSCC.

## Conclusions

Proton radiation is a promising, potentially less toxic alternative to x-ray radiation for HNSCC and may well have enhanced biological effects on tumors relative to x-ray radiation. Based on the known differences between protons and photons and their effects on gene and protein expression patterns, DNA damage and repair, cell death mechanisms, and tumor immune responses, further studies are warranted to investigate the basis for enhanced effects of molecular or immune targeted therapy on the response of HNSCC to proton versus photon radiation. Such studies will facilitate a personalized de-intensification approach with combination therapy that will enable dose de-escalation to normal tissues simultaneously with variable RBE-based dose modifications to tumors, presumably leading to improved tumor control and reduced normal tissue damage for patients with HNSCC.
